# Effects of SGLT2 inhibition on insulin use in CKD and type 2 diabetes: insights from the CREDENCE trial

**DOI:** 10.1093/ndt/gfaf044

**Published:** 2025-02-28

**Authors:** Bryony Beal, Luke Buizen, Emily K Yeung, Lauren Heath, Lauren Houston, David Z I Cherney, Meg Jardine, Carol Pollock, Clare Arnott, Sradha S Kotwal, Hiddo J L Heerspink, Vlado Perkovic, Brendon L Neuen

**Affiliations:** Department of Renal Medicine, Royal North Shore Hospital, Sydney, NSW, Australia; George Institute for Global Health, University of New South Wales, Sydney, NSW, Australia; Department of Nephrology and Transplantation, Guy's and St Thomas’ NHS Foundation Trust, London, UK; Department of Renal Medicine, Royal North Shore Hospital, Sydney, NSW, Australia; George Institute for Global Health, University of New South Wales, Sydney, NSW, Australia; Faculty of Medicine and Health, University of New South Wales, Sydney, NSW, Australia; Department of Medicine, Division of Nephrology, Toronto General Hospital, Toronto, ON, Canada; George Institute for Global Health, University of New South Wales, Sydney, NSW, Australia; NHMRC Clinical Trials Centre, University of Sydney, Sydney, NSW, Australia; Department of Renal Medicine, Concord Repatriation and General Hospital, Sydney, NSW, Australia; Department of Renal Medicine, Royal North Shore Hospital, Sydney, NSW, Australia; Kolling Institute, University of Sydney, Sydney, NSW, Australia; George Institute for Global Health, University of New South Wales, Sydney, NSW, Australia; Faculty of Medicine and Health, University of New South Wales, Sydney, NSW, Australia; Department of Cardiology, Royal Prince Alfred Hospital, Sydney, NSW, Australia; George Institute for Global Health, University of New South Wales, Sydney, NSW, Australia; Department of Renal Medicine, Prince of Wales Hospital, Sydney, NSW, Australia; George Institute for Global Health, University of New South Wales, Sydney, NSW, Australia; Department of Clinical Pharmacy and Pharmacology, University of Groningen, University Medical Centre Groningen, Groningen, The Netherlands; George Institute for Global Health, University of New South Wales, Sydney, NSW, Australia; Faculty of Medicine and Health, University of New South Wales, Sydney, NSW, Australia; Department of Renal Medicine, Royal North Shore Hospital, Sydney, NSW, Australia; George Institute for Global Health, University of New South Wales, Sydney, NSW, Australia

**Keywords:** canagliflozin, CKD, diabetic kidney disease, insulin, SGLT2 inhibitors

## Abstract

**Background:**

Insulin is a mainstay treatment for diabetes, but its use is associated with weight gain and hypoglycaemia. Data on the effects of sodium–glucose co-transporter 2 (SGLT2) inhibitors on insulin use in people with chronic kidney disease (CKD) are limited.

**Methods:**

We conducted a post hoc analysis of the Canagliflozin and Renal Events in Diabetes with Established Nephropathy Clinical Evaluation trial. Effects of canagliflozin versus placebo on insulin use (initiation, dose intensification, reduction and discontinuation) in people with CKD and type 2 diabetes were evaluated using Cox regression models. The primary outcome was insulin initiation or a >25% insulin dose intensification (in those not receiving and receiving insulin at baseline, respectively). Effects on kidney, cardiovascular and safety outcomes by baseline insulin use were also assessed.

**Results:**

Among 4401 participants, 2884 (65.5%) were receiving insulin at baseline; these participants were more likely to have lower estimated glomerular filtration rate, higher albuminuria and a longer duration of diabetes (all *P* < .001). Over a median on-treatment period of 2.0 years, canagliflozin reduced the need for insulin initiation or a >25% dose intensification by 19% compared with placebo {hazard ratio [HR] 0.81 [95% confidence interval (CI) 0.71–0.93]}, irrespective of baseline kidney function or albuminuria (both *P*-interaction > .10). Sustained insulin dose reductions of >50% were achieved more frequently with canagliflozin than placebo [HR 1.49 (95% CI 1.15–1.91)], although no difference in insulin discontinuation was observed between treatment arms. Effects of canagliflozin on kidney, cardiovascular and safety outcomes were consistent regardless of baseline insulin use (all *P*-interaction > .05).

**Conclusions:**

In CKD and type 2 diabetes, canagliflozin reduces insulin use with consistent effects regardless of baseline kidney function. This supports the use of canagliflozin in people with CKD, not only for end-organ protection, but also to improve glycaemic control and reduce exposure to insulin and its associated adverse effects.

KEY LEARNING POINTS **What was known:**

- Insulin is a mainstay treatment for diabetes but associated with hypoglycaemia and weight gain.
- Sodium–glucose co-transporter 2 (SGLT2) inhibitors are guideline recommended for people with chronic kidney disease (CKD) and type 2 diabetes.
- Kidney Disease: Improving Global Outcomes guidelines suggest dose adjustment of other glucose-lowering agents may be required when initiating SGLT2 inhibitors.

**This study adds:**

- Canagliflozin significantly reduces insulin initiation and dose intensification in patients with CKD and type 2 diabetes, with consistent effects regardless of estimated glomerular filtration rate and urine albumin:creatinine ratio.
- In patients receiving insulin, canagliflozin increases the likelihood of achieving clinically meaningful reductions in insulin dose.
- Effects of canagliflozin on cardiovascular, kidney and safety outcomes are consistent irrespective of insulin use.

**Potential impact:**

- This analysis supports the use of canagliflozin in CKD, not only for end-organ protection, but also to limit exposure to insulin and its associated adverse effects.
- These findings should engender confidence among clinicians regarding the use of canagliflozin in CKD, with and without insulin.

## INTRODUCTION

Over the past century, insulin has been a cornerstone therapy for diabetes mellitus. However, it can be a burdensome treatment with several adverse effects. Insulin use requires frequent self-injections, a potential barrier to patient acceptance of therapy. It is also associated with weight gain; the average weight gain after 10 years of insulin therapy is ≈7 kg in patients with type 2 diabetes [1]. Insulin also increases the risk of hypoglycaemic events, which in turn are associated with increased morbidity and mortality [2].

Sodium–glucose co-transporter 2 (SGLT2) inhibitors were initially developed as glucose-lowering agents, with a unique glycosuric effect. SGLT2 inhibitors act at the proximal convoluted tubule to reduce glucose reabsorption [3, 4], with an average reduction in haemoglobin A1c (HbA1c) of 0.5–1% in people with normal kidney function [5–9]. The Kidney Disease: Improving Global Outcomes (KDIGO) 2024 guidelines for the management of chronic kidney disease (CKD) includes a class 1A recommendation for the use of SGLT2 inhibitors in patients with type 2 diabetes, CKD and an estimated glomerular filtration rate (eGFR) >20 ml/min/1.73 m^2^ [10].

The KDIGO guidelines include a specific practice point that other glucose-lowering medications may need to be dose adjusted with the initiation of SGLT2 inhibitors [10]. However, there is minimal guidance for clinicians on the magnitude of effect of SGLT2 inhibitors on insulin dosing in the literature to date, particularly in people with CKD. While the risk of hypoglycaemia with SGLT2 inhibitor monotherapy is low, the risk of hypoglycaemia in patients receiving insulin, particularly those with CKD, is less well defined.

We hypothesized that canagliflozin would reduce insulin initiation and dose intensification in patients with type 2 diabetes and CKD.

We therefore undertook a post hoc analysis of the Canagliflozin and Renal Events in Diabetes with Established Nephropathy Clinical Evaluation (CREDENCE; NCT02065791) trial [11] to evaluate the effects of canagliflozin on insulin use, including dose initiation, intensification, dose reduction and discontinuation, in people with CKD and type 2 diabetes. We also evaluated the effects of canagliflozin on kidney, cardiovascular and safety outcomes, including diabetic ketoacidosis, by baseline insulin use.

## MATERIALS AND METHODS

### Study design

We conducted a post hoc analysis of the CREDENCE trial, a randomized, double-blind, placebo-controlled, event-driven clinical outcome trial. The CREDENCE trial evaluated the effects of canagliflozin versus placebo on a primary composite outcome of doubling of serum creatinine, kidney failure or death due to cardiovascular disease or kidney failure in patients with CKD and type 2 diabetes who were receiving the standard of care, including a maximum tolerated daily dose of an angiotensin-converting enzyme inhibitor or angiotensin II receptor blocker. The trial was conducted at 695 sites across 34 countries. The detailed methods and main findings of this study have been previously published [11]. All patients provided informed consent and ethics approval was obtained at all participating centres.

### Participants

The CREDENCE trial included participants ≥30 years of age, with type 2 diabetes (HbA1c 6.5–12%) and CKD, with a baseline eGFR of 30–<90 ml/min/1.73 m^2^ and a urine albumin:creatinine ratio (UACR) ≥300–5000 mg/g. All participants were required to be established on renin–angiotensin system blocker for 4 weeks prior to randomization. Participants with type 1 diabetes, non-diabetic CKD (including autosomal dominant polycystic kidney disease), uncontrolled hypertension (systolic blood pressure >180 mmHg or diastolic blood pressure >100 mmHg) or a history of dialysis or transplantation were excluded, as well as those currently on a mineralocorticoid receptor antagonist or on immunosuppression for kidney disease.

### Randomization

All participants underwent a 2-week, single-blind, placebo run-in period, following which they were randomized 1:1 to canagliflozin 100 mg once daily or matching placebo. Randomization occurred via a centralized computer-based schedule, stratified based on the pre-randomization eGFR.

### Outcomes and follow-up

The primary outcome in this post hoc analysis was a composite of insulin initiation or dose intensification (defined as a >25% increase in insulin dose). Initiation of insulin was assessed in participants not receiving insulin at baseline, while dose intensification was assessed in participants receiving insulin at baseline. In participants receiving insulin at baseline, dose intensification was evaluated using a range of different thresholds of insulin dose titration, including a >25% dose increase, a sustained >25% dose increase, a >50% dose increase and a sustained >50% dose increase. In participants receiving insulin at baseline, we also evaluated effects on insulin dose reduction, including a >25% dose reduction, a sustained >25% dose reduction, a >50% dose reduction and a sustained >50% dose reduction, as well as insulin discontinuation. Sustained insulin intensification or dose reduction was defined as a persistent dose increase or decrease for >28 days. We also assessed the effects of canagliflozin on clinical outcomes by baseline insulin use. Clinical outcomes included doubling of serum creatinine, kidney failure or death due to kidney failure, heart failure hospitalization or cardiovascular death, non-fatal myocardial infarction, non-fatal stroke or cardiovascular death. Insulin dosing information was obtained using concomitant medication usage data recorded at each study visit during the trial. Patients were censored at the point of death, point of last contact with the trial investigators or the end of the double-blind on-treatment period, whichever occurred first. The end of the on-treatment period was chosen as the point of censoring due to the inconsistent recording of concomitant medication use after this date [12].

### Statistical analysis

All randomized trial participants were included in this post hoc analysis. Characteristics of participants according to baseline use of insulin were compared using *t*-tests for normally distributed continuous variables and chi-squared tests for categorical variables. Continuous variables were reported as mean and standard deviation (SD) and categorical variables were reported as frequency and percentage.

Cox proportional hazards regression models were used to assess the effect of canagliflozin on insulin initiation or intensification in the intention-to-treat population. We included a stratification term for the category of eGFR at screening (30–<45 ml/min/1.73 m^2^, 45–<60 ml/min/1.73 m^2^ or 60–<90 ml/min/1.73 m^2^), as was prespecified in the original trial publication [11]. Treatment effects were expressed as hazard ratios (HRs) with corresponding 95% confidence intervals (CIs).

Because of differences in baseline characteristics between participants receiving and not receiving insulin at baseline, we conducted a sensitivity analysis adjusting for differences in these baseline characteristics, including the use of other concomitant glucose-lowering agents.

Effects of canagliflozin on kidney, cardiovascular and safety outcomes by baseline insulin use were also evaluated using Cox regression models with treatment by subgroup interaction terms included in the relevant models.

Because the glycosuric effect of SGLT2 inhibitors is attenuated in people with CKD, we assessed the consistency of treatment effect on the primary outcome of insulin initiation or dose intensification across the spectrum of baseline GFR and UACR, with results displayed using restricted cubic splines.

All analyses were performed using SAS enterprise guide version (7.1) (SAS Institute, Cary, NC, USA) and Stata version (v18) (StataCorp, College Station, TX, USA).

## RESULTS

### Baseline characteristics

The CREDENCE trial randomized 4401 participants with CKD and type 2 diabetes from March 2014 to May 2017. A total of 2884 participants (65.5%) were on insulin at baseline, 1452 in the canagliflozin arm and 1432 in the placebo arm. Participants receiving insulin therapy at baseline were more likely to be female, have a longer duration of diabetes and have a higher baseline HbA1c (Table 1). Insulin-treated participants were also more likely to have a history of cardiovascular disease, a higher baseline body mass index, lower eGFR and higher levels of albuminuria (Table 1). At baseline, there was greater use of other glucose-lowering agents, including metformin, sulfonylureas and glucagon-like peptide-1 receptor (GLP-1) agonists, in the non-insulin-treated group (Table 1). Characteristics of participants randomized to canagliflozin and placebo were generally well balanced in each insulin subgroup (Supplementary Table S1).

**Table 1:** Characteristics of participants receiving and not receiving insulin at baseline.

	CREDENCE population (*N* = 4401)		
Characteristics	Insulin (*n* = 2884)	No insulin (*n* = 1517)	*P*-value
Age (years), mean (SD)	62.5 (9.2)	63.7 (9.2)	<.001
Female, *n* (%)	1027 (35.6)	467 (30.8)	.001
Race, *n* (%)			
White	1870 (64.8)	1061 (69.9)	<.001
Black/African American	172 (6.0)	52 (3.4)	
Asian	558 (19.3)	319 (21.0)	
Other	284 (9.8)	85 (5.6)	
Diabetes duration (years), mean (SD)	17.5 (8.4)	12.6 (8.2)	<.001
BMI (kg/m^2^), mean (SD)	31.9 (6.4)	30.3 (5.6)	<.001
Baseline HbA1c (%), mean (SD)	8.4 (1.3)	8.0 (1.3)	<.001
Systolic BP (mmHg), mean (SD)	140.9 (15.9)	138.3 (14.8)	<.001
History of CV disease, *n* (%)	1512 (52.4)	708 (46.7)	<.001
History of heart failure, *n* (%)	389 (13.5)	263 (17.3)	<.001
eGFR (ml/min/1.73 m^2^)			
Mean (SD)	54.3 (17.7)	59.7 (18.8)	<.001
<45	981 (34.0)	384 (25.3)	<.001
45–<60	867 (30.1)	399 (26.3)	
≥60	1036 (35.9)	733 (48.3)	
UACR, *n* (%)			
Median (25th–75th percentile)	983.5 (496.0–1954.5)	805.0 (421.0–1601.0)	<.001
>1000 mg/g	1421 (49.3)	632 (41.7)	<.001
≤1000 mg/g	1463 (50.7)	885 (58.3)	
Glucose-lowering agents, *n* (%)			
Metformin	1361 (47.2)	1184 (78.0)	<.001
Sulphonylurea	372 (12.9)	896 (59.1)	<.001
GLP-1RA	138 (4.8)	45 (3.0)	.004

BMI: body mass index; BP: blood pressure; CV: cardiovascular; GLP-1RA: glucagon-like peptide-1 receptor agonist.

### Effects of canagliflozin on insulin initiation and dose intensification

**Figure 1:**
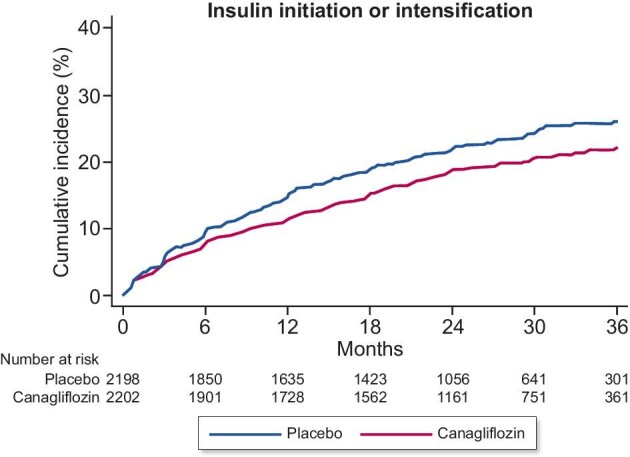
Effect of canagliflozin versus placebo on insulin initiation or a>25% dose intensification.

**Figure 2:**
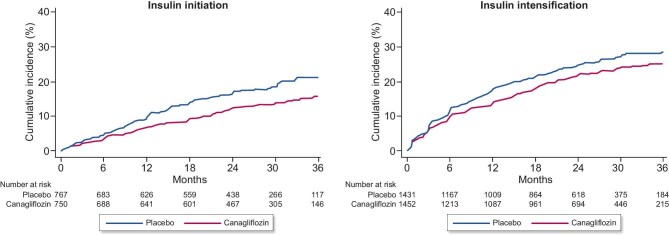
Effect of canagliflozin versus placebo on insulin initiation and a >25% dose intensification.

Over a median on-treatment period of 2.0 years, insulin initiation or dose intensification by >25% was required in 407/2202 (18.5%) participants in the canagliflozin arm and 476/2199 (21.6%) participants in the placebo arm. Canagliflozin reduced the occurrence of the primary outcome of insulin initiation or a >25% insulin dose intensification by 19% compared with placebo [HR 0.81 (95% CI 0.71–0.93); Fig. 1]. Among the 1517 (34.5%) participants who were insulin naïve at randomization, canagliflozin reduced the need for insulin initiation by 28% [HR 0.72 (95% CI 0.55–0.93); Fig. 2]. Of the 2884 (65.5%) participants on insulin at baseline, treatment with canagliflozin reduced the need for insulin dose intensification by 16% [HR 0.84 (95% CI 0.72–0.98); Fig. 2].

**Figure 3:**
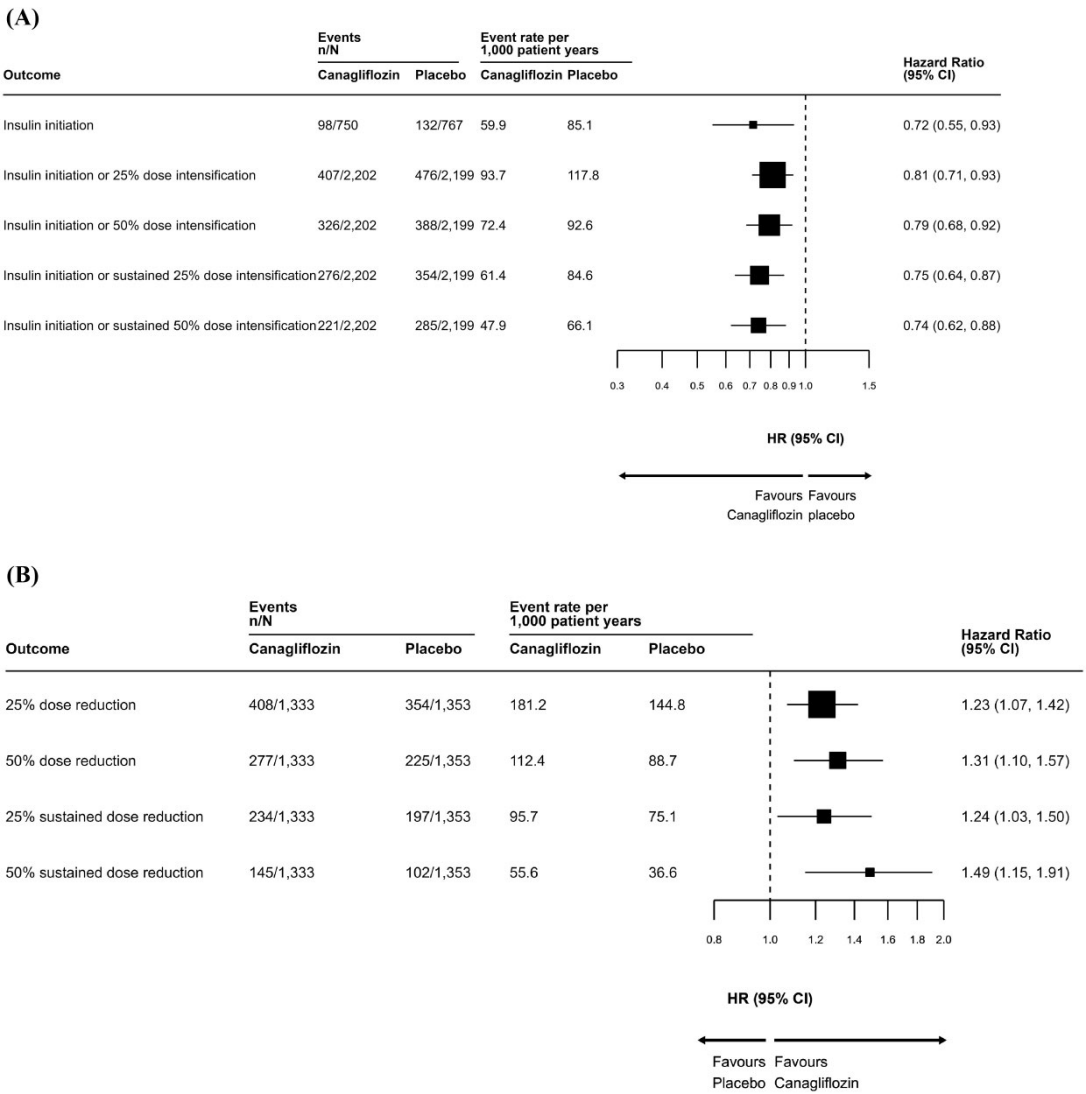
Effects of canagliflozin versus placebo on **(A)** insulin initiation or dose intensification and **(B)** insulin dose reduction based on different dose thresholds and for changes to be sustained or unsustained.

Reductions in insulin initiation or intensification with canagliflozin were observed regardless of the threshold used to define dose intensification and irrespective of whether dose changes were sustained or unsustained (Fig. 3A).

**Figure 4:**
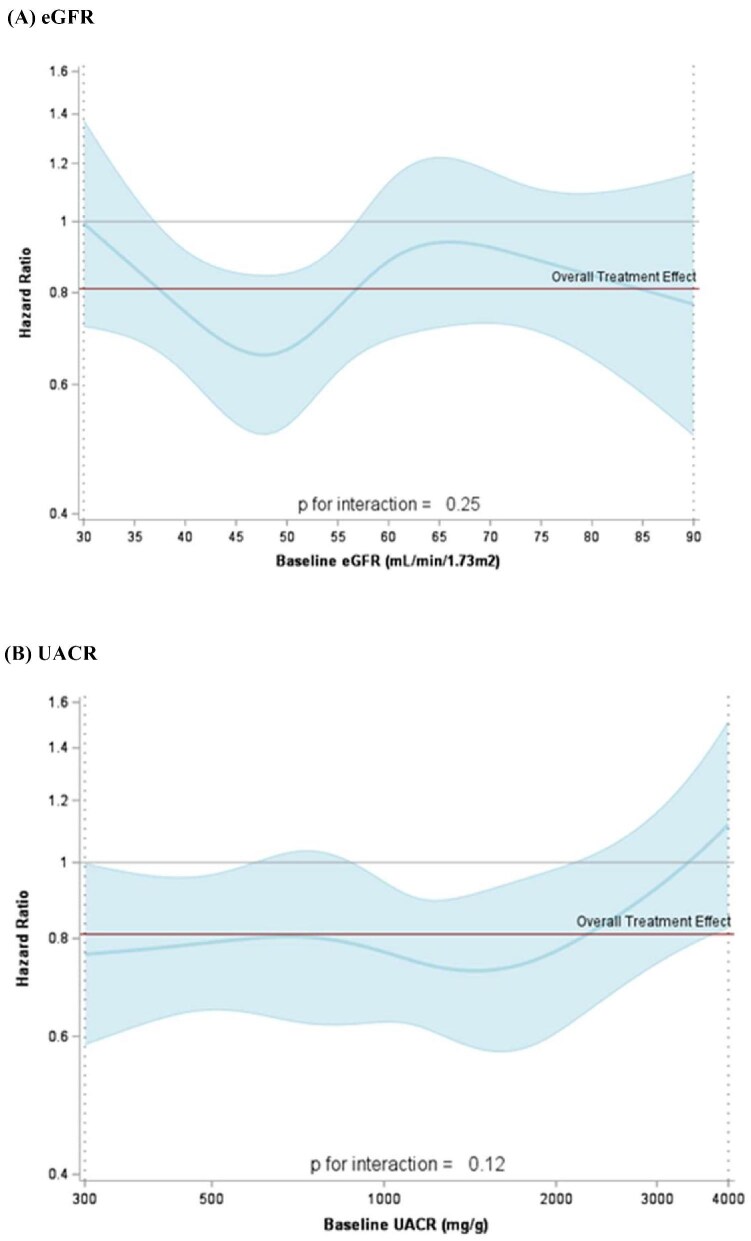
Effect of canagliflozin versus placebo on insulin initiation or a >25% dose increase by baseline **(A)** eGFR and **(B)** UACR.

The effect of canagliflozin on insulin initiation or a>25% dose intensification was consistent regardless of baseline eGFR (*P*-interaction = .25; Fig. 4A) and baseline albuminuria (*P*-interaction = .12; Fig. 4B). Results were similarly consistent when eGFR and UACR were fitted categorically (Supplementary Table S2).

The effect on the primary outcome was consistent in sensitivity analyses adjusting for differences in baseline characteristics between participants using insulin and not using insulin at baseline [HR 0.82 (95% CI 0.72–0.93)].

### Effect of canagliflozin on insulin dose reduction and discontinuation

In participants receiving insulin at baseline, sustained dose reductions of >50% were achieved more frequently with canagliflozin than placebo [HR 1.49 (95% CI 1.15–1.91)]. This effect was observed regardless of the threshold used to define dose reduction and irrespective of whether dose changes were sustained or unsustained (Fig. 3B). There were few people who discontinued insulin throughout the on-treatment period: 52 (3.6%) in the canagliflozin arm and 57 (4.0%) in the placebo arm. Canagliflozin did not affect the need for insulin discontinuation [HR 0.88 (95% CI 0.60–1.28)].

### Effects of canagliflozin on kidney, cardiovascular and safety outcomes by baseline insulin use

**Figure 5:**
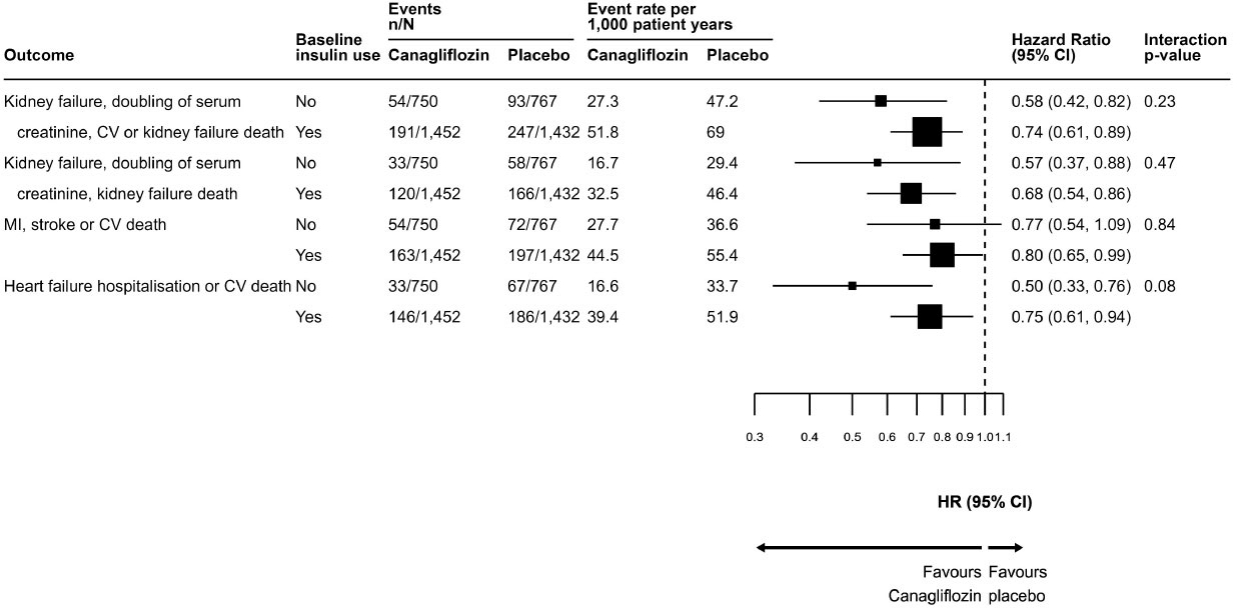
Effect of canagliflozin versus placebo on kidney and cardiovascular outcomes by baseline insulin use. CV: cardiovascular; MI: myocardial infarction.

The effects of canagliflozin on kidney and cardiovascular outcomes are presented in Fig. 5. Event rates for all cardiovascular and kidney outcomes were higher for participants receiving insulin compared with those who were not on insulin at baseline. For all outcomes, relative risk reductions with canagliflozin were consistent regardless of insulin use at baseline.

Similarly, effects on key safety outcomes, including hypoglycaemia, were not modified by insulin use (all *P*-interaction > .05; Table 2). A total of 12 participants experienced ketoacidosis, 11 of whom were randomized to canagliflozin. Of the 12 participants, 11 (91.7%) were receiving insulin at baseline. Four participants experienced both insulin intensification and ketoacidosis, and two of these cases had undergone insulin intensification prior to experiencing ketoacidosis.

**Table 2:** Effects of canagliflozin on safety outcomes by baseline insulin use.

Outcome	Insulin use at baseline	Canagliflozin, *n*/*N* (*N* = 2202)	Placebo, *n*/*N* (*N* = 2199)	HR (95% CI)	*P*-interaction value
Serious adverse events	Yes	546/1452	572/1432	0.90 (0.80, 1.02)	.28
	No	191/750	234/767	0.80 (0.66, 0.97)	
Hypoglycaemia	Yes	192/1452	208/1432	0.89 (0.73, 1.09)	.56
	No	33/750	32/767	1.04 (0.64, 1.70)	
Volume depletion	Yes	95/1452	84/1432	1.10 (0.82, 1.47)	.14
	No	49/750	31/767	1.66 (1.06, 2.60)	
AKI	Yes	68/1452	79/1432	0.82 (0.59, 1.13)	.69
	No	18/750	19/767	0.96 (0.50, 1.82)	
UTI	Yes	184/1452	162/1432	1.10 (0.89, 1.35)	.75
	No	61/750	59/767	1.01 (0.71, 1.45)	
Mycotic genital infection: male	Yes	19/920	2/937	9.71 (2.26, 41.67)	.93
	No	9/520	1/530	8.59 (1.09, 67.87)	
Mycotic genital infection: female	Yes	16/532	6/495	2.45 (0.96, 6.26)	.57
	No	6/230	4/237	1.53 (0.43, 5.44)	

AKI: acute kidney injury; UTI: urinary tract infection.

## DISCUSSION

In the CREDENCE trial, treatment with canagliflozin resulted in clinically meaningful reductions in insulin initiation or dose intensification, irrespective of the threshold used to define dose intensification and whether these changes in dose were sustained or unsustained. In participants receiving insulin at baseline, significantly more achieved clinically meaningful reductions in insulin dose; however, no significant impact was demonstrated on insulin discontinuation. Importantly, while the glycosuric effects of SGLT2 inhibition are directly proportional to GFR, reductions in insulin use were consistent across the spectrum of kidney function down to an eGFR of 30 ml/min/1.73 m^2^. These data highlight the beneficial impacts of canagliflozin on insulin dosing in patients with CKD and type 2 diabetes.

Long-term randomized data on the impacts of SGLT2 inhibitors on insulin use in people with CKD are limited. In the EMPA-REG OUTCOME trial (NCT01131676), empagliflozin decreased insulin initiation by 60% and dose intensification by 20%, with similar results reported with ertugliflozin in VERTIS CV (NCT01986881) [13–16]. Similar benefits were observed in the EMPEROR (NCT03057951 and NCT03057977), DAPA-HF (NCT03036124) and DELIVER (NCT03619213) trials, which enrolled participants with type 2 diabetes and heart failure; however, these populations mostly had preserved kidney function [17, 18]. A secondary analysis of the DAPA-CKD (NCT03036150) trial demonstrated that dapagliflozin decreased insulin initiation throughout the trial follow-up period [19]. Our analysis of the data from CREDENCE adds to the existing literature, extending previous findings to people with established CKD, demonstrating consistency of effects across a spectrum of eGFR and UACR, as well as quantifying the likelihood of achieving clinically meaningful reductions in insulin dose.

SGLT2 inhibitors act by reducing the reabsorption of glucose at the level of the proximal convoluted tubule. As GFR decreases, however, the amount of filtered glucose delivered to the proximal tubule similarly decreases, resulting in a reduction in the glycosuric effect of SGLT2 inhibitors at lower levels of kidney function [20]. We hypothesized that, like the effect on HbA1c, any effect of canagliflozin on insulin use would be attenuated in participants with reduced kidney function. However, we observed no evidence of effect modification by eGFR. SGLT2 inhibitors may improve beta cell function and proliferation and peripheral insulin sensitivity and contribute to weight loss and a reduction in tissue inflammation, all of which may improve glycaemic control independent of the increased urinary glucose excretion effect of SGLT2 inhibitors [9, 21–23]. While the observed relative risk reduction in insulin initiation or intensification was more modest compared with that seen in other SGLT2 inhibitor trials, even moderate effects remain clinically relevant given the limited glucose-lowering options available in advanced CKD and the adverse effects associated with insulin use.

The KDIGO diabetes guidelines provide a practice point suggesting that clinicians may need to consider dose adjustment of other hypoglycaemic agents to facilitate the commencement of SGLT2 inhibitors [10]. The observed reductions in insulin dose requirements in our analysis reinforces this recommendation. While it is recognized that SGLT2 inhibitors rarely cause hypoglycaemia as monotherapy, this risk may be increased with concomitant insulin use. In this context, our analysis also provides important data reinforcing the safety of SGLT2 inhibitors when used with and without concomitant insulin.

The CREDENCE trial was a well-designed international multicentre randomized controlled trial, with thorough recording of concomitant medication use during the on-treatment period. This enabled accurate assessment of insulin commencement or dose adjustments required in the participants throughout the trial period. The large sample size allowed for a reliable assessment of the effects of canagliflozin on a range of insulin-based outcomes, with the full intention-to-treat population able to be utilized for this analysis. The inclusion of participants with low baseline eGFR and high levels of albuminuria allowed evaluation of the consistency of the effect across these clinically important markers of kidney function.

However, there are limitations that must be recognized. This was a post hoc analysis and CREDENCE was not specifically designed to assess effects on insulin initiation or dose intensification. Patients with an eGFR <30 ml/min/1.73 m^2^ were not enrolled in the CREDENCE trial and whether these effects on insulin use are generalizable to patients with advanced CKD is not known. This analysis leveraged concomitant medication data from the trial, recorded at each study visit, which may have been variably recorded between trial sites and was not centrally adjudicated. Additionally, due to variable collection of concomitant medication data following completion of the on-treatment period, we were required to censor participants at this point in the follow-up, potentially reducing the power to detect longer-term treatment effects. However, our findings are consistent with those reported in the existing literature, strengthening the robustness of our conclusions.

In summary, in people with CKD and type 2 diabetes, treatment with canagliflozin resulted in clinically meaningful reductions in insulin use. These findings support the use of canagliflozin in people with CKD not only for end-organ protection, but also to limit exposure to insulin therapy and the risk of its associated adverse effects.

## Supplementary Material

gfaf044_Supplemental_File

## Data Availability

Data from this study are available in the public domain via the Yale University Open Data Access Project (http://yoda.yale.edu/).
