# Supplementary material for: Effects of SGLT2 inhibition on insulin use in CKD and type 2 diabetes: insights from the CREDENCE trial
Source: Nephrol Dial Transplant. 2025 Feb 28;40(9):1727–35. doi: 10.1093/ndt/gfaf044 (PMC12394126; doi:10.1093/ndt/gfaf044)
Supplement: gfaf044_Supplemental_File [file gfaf044_supplemental_file.docx]

**Effects of SGLT2 inhibition on insulin use in CKD and type 2 diabetes: Insights from the CREDENCE trial**

**Supplementary appendix**

Table S1: Characteristics of participants randomised to canagliflozin and placebo by baseline insulin use.

Table S2: Subgroup analysis of effect of canagliflozin on insulin initiation or >25% dose intensification stratified by baseline eGFR and UACR.

**Table S1: Characteristics of participants randomised to canagliflozin and placebo by baseline insulin use.**

| **Characteristic** | **CREDENCE population**  **N = 4401** | | | |
| --- | --- | --- | --- | --- |
|  | Insulin  N= 2884 | | No insulin  N= 1517 | |
|  | Canagliflozin  N= 1452 | Placebo  N= 1432 | Canagliflozin  N= 750 | Placebo  N= 767 |
| Age, mean (SD), years | 62.3 (9.0) | 62.6 (9.3) | 63.7 (9.4) | 63.8 (9.0) |
| Female, n (%) | 532 (36.6) | 495 (34.6) | 230 (30.7) | 237 (30.9) |
| Race, n (%)  White  Black/African American  Asian  Other | 949 (65.4)  90 (6.2)  269 (18.5)  144 (9.9) | 921 (64.3)  82 (5.7)  289 (20.2)  140 (9.8) | 538 (71.7)  22 (2.9)  156 (20.8)  34 (4.5) | 523 (68.2)  30 (3.9)  163 (21.3)  51 (6.6) |
| Diabetes duration, mean (SD), years | 17.2 (8.5) | 17.7 (8.2) | 12.3 (8.1) | 12.8 (8.3) |
| BMI, mean (SD), kg/m2 | 31.8 (6.4) | 31.9 (6.4) | 30.4 (5.5) | 30.2 (5.6) |
| Baseline HbA1c, mean (SD), % | 8.4 (1.3) | 8.4 (1.3) | 7.9 (1.3) | 8.0 (1.3) |
| Systolic BP, mean (SD), mmHg | 140.7 (15.9) | 141.1 (15.9) | 138.1 (14.8) | 138.4 (14.9) |
| History of CV disease, n (%) | 761 (52.4) | 751 (52.4) | 352 (46.9) | 356 (46.4) |
| History of heart failure, n (%) | 208 (14.3) | 181 (12.6) | 121 (16.1) | 142 (18.5) |
| eGFR (mL/min/1.73m2)  Mean (SD)  <45  45-<60  ≥ 60 | 54.2 (17.6)  499 (34.4)  437 (30.1)  516 (35.5) | 54.4 (17.8)  482 (33.7)  430 (30.0)  520 (36.3) | 60.5 (18.6)  179 (23.9)  193 (25.7)  377 (50.3) | 58.9 (19.0)  205 (26.7)  206 (26.9)  356 (46.4) |
| UACR, n (%)  Median (25^th^. 75^th^ centile)  >1000mg/g  ≤ 1000mg/g | 960.5 (470.5, 1949.0)  704 (48.5)  748 (51.5) | 1003.5 (520.0, 1971.5)  717 (50.1)  715 (49.9) | 826.0 (438.0, 1620.0)  313 (41.7)  437 (58.3) | 774.0 (403.0, 1588.0)  319 (41.6)  448 (58.4) |
| Glucose lowering agents, n (%)  Metformin  Sulphonylurea  GLP-1 RA | 684 (47.1)  172 (11.8)  65 (4.5) | 677 (47.3)  200 (14.0)  73 (5.1) | 592 (78.9)  440 (58.7)  24 (3.2) | 592 (77.2)  456 (59.5)  21 (2.7) |

SD: standard deviation; BMI: body mass index; BP: blood pressure; CV: cardiovascular; eGFR: estimated glomerular filtration rate; UACR: urinary albumin-creatinine ratio; GLP-1 RA: glucagon-like peptide-1 receptor agonist.

**Table S2: Subgroup analysis of effect of canagliflozin on insulin initiation or >25% dose intensification stratified by baseline eGFR and UACR.**

| Characteristic | CREDENCE population (n = 4401) | | | |
| --- | --- | --- | --- | --- |
|  | Canagliflozin  n = 2202 | Placebo  n = 2199 | HR  (95% CI) | P value for interaction |
| All | 407/2022 | 476/2199 | 0.81 (0.71-0.93) |  |
| eGFR (ml/min/1.73m2)  <45  45-<60  ≥60 | 142/678  128/630  137/893 | 166/687  150/636  160/876 | 0.82 (0.66-1.03)  0.82 (0.65-1.04)  0.80 (0.64-1.01) | 0.990 |
| UACR (mg/g)  ≤ 1000  >1000 | 204/1185  203/1017 | 241/1163  235/1036 | 0.78 (0.65-0.95)  0.85 (0.70-1.02) | 0.540 |

eGFR: estimated glomerular filtration rate; UACR: urinary albumin-creatinine ratio; HR: hazard ratio; CI: confidence interval.
